# Dysbiosis of intestinal microbiota mediates tubulointerstitial injury in diabetic nephropathy via the disruption of cholesterol homeostasis

**DOI:** 10.7150/thno.40571

**Published:** 2020-02-03

**Authors:** Ze Bo Hu, Jian Lu, Pei Pei Chen, Chen Chen Lu, Jia Xiu Zhang, Xue Qi Li, Ben Yin Yuan, Si Jia Huang, Xiong Zhong Ruan, Bi Cheng Liu, Kun Ling Ma

**Affiliations:** 1 Institute of Nephrology, Zhongda Hospital, School of Medicine, Southeast University, Nanjing, 210009, China.; 2 Department of Pathophysiology, School of Basic Medicine, Wannan Medical College, Wuhu, 241002, China.; 3 Centre for Nephrology, University College London (UCL) Medical School, Royal Free Campus, UK.

**Keywords:** diabetic nephropathy, gut microbiota, acetate, cholesterol homeostasis, tubulointerstitial injury

## Abstract

**Background**: Our previous study demonstrated that the disruption of cholesterol homeostasis promotes tubulointerstitial injury in diabetic nephropathy (DN). This study aimed to further investigate the effects of gut microbiota dysbiosis on this process and explored its potential mechanism.

**Methods**: Diabetic rats treated with broad-spectrum oral antibiotics or faecal microbiota transplantation (FMT) from the healthy donor group and human kidney 2 (HK-2) cells stimulated with sodium acetate were used to observe the effects of gut microbiota on cholesterol homeostasis. The gut microbiota distribution was measured by 16S rDNA sequencing with faeces. Serum acetate level was examined by gas chromatographic analysis. Protein expression of G protein coupled receptor 43 (GPR43) and molecules involved in cholesterol homeostasis were assessed by immunohistochemical staining, immunofluorescence staining, and Western Blotting.

**Results**: Depletion of gut microbiota significantly attenuated albuminuria and tubulointerstitial injury. Interestingly, serum acetate levels were also markedly decreased in antibiotics-treated diabetic rats and positively correlated with the cholesterol contents in kidneys. An *in vitro* study demonstrated that acetate significantly increased cholesterol accumulation in HK-2 cells, which was caused by increased expression of proteins mainly modulating cholesterol synthesis and uptake. As expected, FMT effectively decreased serum acetate levels and alleviated tubulointerstitial injury in diabetic rats through overriding the disruption of cholesterol homeostasis. Furthermore, GPR43 siRNA treatment blocked acetate-mediated cholesterol homeostasis dysregulation in HK-2 cells through decreasing the expression of proteins governed cholesterol synthesis and uptake.

**Conclusion**: Our studies for the first time demonstrated that the acetate produced from gut microbiota mediated the dysregulation of cholesterol homeostasis through the activation of GPR43, thereby contributing to the tubulointerstitial injury of DN, suggesting that gut microbiota reprogramming might be a new strategy for DN prevention and therapy.

## Introduction

It is widely accepted that diabetic nephropathy (DN) is a disease mainly characterized by glomerular injury. Progressive glomerulosclerosis is the main cause of renal failure. However, Dalla Vestra *et al.* 1 recently reported that only 30% of diabetic mellitus (DM) patients have typical glomerular lesions, up to 40% of DM patients have tubulointerstitial lesions and renal vascular lesions, and the remaining 30% of DM patients have a normal renal structure. This finding reveals that renal interstitial injury plays an important role in the progression of DN. Furthermore, the degree of tubulointerstitial injury is closely correlated with the prognosis of DN 2.

The mechanism of tubulointerstitial injury in DN is still unclear. Maezawa *et al.* reported that tubuleinterstitial damage is a secondary effect of glomerular protein leakage induced by hyperglycemia in the progression of DN 3. Artunc *et al.* indicated that insulin resistance affects renal haemodynamics and tubular function, resulting in tubulointerstitial damage, sodium retention, and arterial hypertension 4. On the other hand, persistent increased uremia toxin in turn contributes to the muscle weakness and muscle wasting, thereby aggravating insulin resistance in chronic kidney disease (CKD) 5. Li *et al*. demonstrated that tubular damage was alleviated in db/db mice treated with bone morphogenetic protein 7 though suppressing multiple inflammatory signaling pathways, suggesting inflammation also play important roles in diabetic tubulopathy 6. Garagliano *et al*. indicated that advanced glycation end products enhance proximal tubular angiotensinogen expression via ERK1/2 in cultured proximal tubular cells, which could be involved in the modulation of tubular cell injuries 7. However, clinical studies have shown that traditional therapies that decrease glucose levels and inhibit RAS activation and inflammation could not completely control the progression of kidney injury in DN 8-10. These findings suggest that in addition to traditional risk factors, some other risk factors could be involved in the pathogenesis of DN as well.

Recent studies reported that the gut microbiota has crosstalk with the kidney and participates in the kidney injury 11, 12. Karlsson *et al.* revealed that a significant decrease in *Rothia* and *Faecalibacterium prausnitzi* abundance in gut microbiota was correlated with insulin resistance in European women with type 2 diabetes 13. Giongo *et al.* showed that the abundance of *Bacteroidetes* was decreased, while *Firmicutes* was increased in young children with type 1 diabetes 14. Yang *et al*. reported that gut *Bacteroides acidifaciens* prevented obesity and improved insulin sensitivity in mice 15. Cani *et al.* also showed that improvement of gut microbiota dysbiosis by *Bifidobacterium* supplementation corrected impaired glucose tolerance and attenuated insulin resistance in diabetic mice 16. These studies suggest that decreased *Bacteroidetes* in diabetes mellitus may indirectly exacerbate insulin resistance. Current use of modulating therapies of the intestinal flora by prebiotics or probiotics have not yet given conclusive results applicable to the clinic 17, 18 although these strategies in human clinical studies have shown some efficacy 19. In addition, Leustean *et al*. reported that intestinal microbiota dysbiosis affected insulin signal and glucose metabolism through inducing insulin resistance, inflammation, and metabolic toxicity 20. Tao *et al.* demonstrated that gut microbiota composition was correlated with the occurrence of DN 21. These studies suggest that gut microbiota dysbiosis may play important roles in the pathogenesis of diabetes. DN is a severe chronic complication of DM with high rates of morbidity and mortality. However, the effects of gut microbiota dysbiosis on the progression of DN have not been fully elucidated.

Lipid metabolic disorder is one of the main complications in DN, which further exacerbates the progression of DN 22. Lipid metabolic disorder is mainly caused by the disruption of cholesterol homeostasis in resident kidney cells. Generally, cholesterol homeostasis is governed by the cholesterol influx and cholesterol efflux pathways. The low-density lipoprotein receptor (LDLr) pathway mainly controls native LDL uptake. Scavenger receptors (CD36, CXC chemokine ligand 16 (CXCL16), etc.) mainly modulate oxidative LDL uptake, and 3-hydroxy-3-methylglutaryl coenzyme A reductase (HMGR) is a rate-limiting enzyme that controls endogenous cholesterol synthesis. Once these lipoprotein pathways are disrupted, cholesterol homeostasis is disrupted. Our previous studies demonstrated that inflammation accelerated renal tubulointerstitial lesions in diabetic mice through the activation of CXCL16 pathway 23. Activation of renin-angiotensin system (RAS) had synergistic effects with hyperlipidemia in accelerating tubulointerstitial injury 24. These findings suggest that dysregulation of cholesterol homeostasis might also be involved in the tubulointerstitial injury of DN.

Therefore, this study aimed to investigate whether gut microbiota dysbiosis mediates the disruption of cholesterol homeostasis and to explore its potential mechanism in tubulointerstitial injury of DN.

## Methods

### Animal studies

Male Sprague-Dawley rats weighing 200-230 g were purchased from Shanghai Bikai Company. All experimental procedures on rats were approved by the ethics review committee of Southeast University. The diabetic model was established in rats by a single dose of intraperitoneal injection of 60 mg/kg streptozotocin (STZ) (Sigma, St Louis, USA) as described previously 25. Rats in the control group were intraperitoneally injected with a single dose of 0.1 mol/L sodium citrate buffer.

The rats were then divided into three groups: the control group treated with drinking water, the DM group treated with drinking water, and the DM+AB group treated with an antibiotic cocktail containing five types of broad-spectrum, poorly absorbable oral antibiotics (0.5g/L vancomycin, 1g/L neomycin, 1g/L metronidazole, 0.1g/L amphotericin B, 1g/L ampicillin) to eradicate the gut microbiota. All rats had free access to food and drinking water/antibiotic cocktails and were raised for another eight weeks. Faecal samples of rats in three groups were collected and stored at -80 °C for 16S ribosome DNA sequencing.

For the faecal microbiota transplantation (FMT) model, diabetic rats were transplanted with faecal microbiota extracted from healthy control rats by gavage at the eighth week after diabetes induction by STZ injection. The rats were then divided into three groups after FMT: the control group, DM group, and FMT group. Fresh faecal pellets from the control rats were collected and immediately added to precooled sterile phosphate buffered saline solution containing 10% glycerol. The faeces were homogenized and pooled in a single sample, filtered with a stainless-steel mesh (0.25 mm), and stored at -80 °C until use. Before the administration of faecal materials, all recipient diabetic rats were given an intravenous tail injection of omeprazole for 3 days to reduce the secretion of gastric acid and allow the survival of microbiota through the stomach. Every recipient diabetic rat accepted 12 ml of faecal microbiota solution by gavage once a day for 3 days. Three days after FMT, fresh faecal samples were collected and stored at -80 °C for 16S ribosome DNA sequencing to estimate the effect of FMT. The rats were then raised for another 8 weeks with free access to food and drinking water.

Body weights were recorded and urine samples were collected before the rats were sacrificed. At termination, blood samples were obtained for biochemical assays. Kidneys were weighed after removing the anadesma on its surface when rats were sacrificed, sliced and stored for various examinations.

### Sequencing of the 16S ribosomal DNA

Fresh faecal pellets were collected on each sampling day and stored at -80 °C until DNA extraction. DNA from 200 mg samples was isolated using the QIAamp DNA Stool Mini Kit (QIAGEN, Hilden, Germany). DNA of the bacterial 16S genes was amplified using general bacterial primers (515F 5'-GTGCCAGCMGCCGCGGTAA-3' and 926R 5'-CCGTCAATTCMTTTGAGTTT-3'). After the purification and quantification of PCR products, the libraries were sequenced by 2*300 bp paired-end sequencing on the MiSeq platform using a MiSeq v3 Reagent Kit (Illumina, San Diego, USA). Then, sequences were quality-filtered by Trimmomatic and merged by FLASH to obtain optimized sequences. The optimized sequences were clustered at 97% sequence identity into operational taxonomic units (OTUs) using the UPARSE pipeline. The OTU representative sequences were analysed for taxonomy by the RDP Classifier algorithm against the Silva (SSU123) 16S rRNA database using a confidence threshold of 70%.

### Periodic acid-Schiff (PAS) staining and transmission electron microscopy

Kidney tissues were immersed into 4% paraformaldehyde and embedded in paraffin for histological observation. Then, 3 μm paraffin sections were stained with PAS solution at room temperature.

Kidney tissues were fixed with 2.5% glutaraldehyde for ultra-microstructure observation of proximal tubular epithelial cells in kidneys. The samples were immersed in 1% osmium tetroxide and then dehydrated with a graded series of acetone before being embedded and polymerized. Ultrathin sections (50-70 nm) were made, stained with uranyl acetate and lead citrate, and observed with a transmission electron microscope (Hitachi, Tokyo, Japan) at 80 kV.

### Immunohistochemical and immunofluorescence staining

For immunohistochemical staining, 3 μm paraffin sections were placed in 10 mM sodium citrate buffer (pH=6.0) and heated in a microwave oven or pressure cooker for antigen unmasking after deparaffinization and rehydration. Then, the sections were blocked with 3% hydrogen peroxide and 5% bovine serum albumin. Next, the sections were reacted with primary antibodies against LDLr (1:200 dilution, Abcam, Cambridge, UK), HMGR (1:200 dilution, Bioss, Beijing, China), CD36 (1:400 dilution, Novus, Colorado, USA), CXCL16 (1:100 dilution, Bioss, Beijing, China ) and G protein coupled receptor 43 (GPR43) (1:200 dilution, Bioss, Beijing, China) overnight at 4 °C and subsequently incubated with biotin-labelled secondary antibodies. Finally, the sections were stained with diaminobenzidine and then discontinued in water when a brown colour was detected.

For immunofluorescence staining, cells were fixed and then treated with 0.02% Triton X-100 for 15 min followed by blocking with 5% BSA for 1 h. Next, the cells were incubated with primary antibodies against LDLr, HMGR, CD36, CXCL16 and GPR43 overnight at 4 °C, followed by treatment with secondary antibodies conjugated to Alexa Fluor 555 for 1 h. Nuclei were stained with 4,6-diamidino-2-phenylindole. Finally, cells were examined by Olympus fluorescence microscopy.

### Blood and urine measurements

Serum levels of glucose, triglycerides (TG), total cholesterol (TC), high-density lipoprotein (HDL) and low-density lipoprotein (LDL) and urine level of creatinine were measured by automatic analysers (Hitachi). Albumin (LifeSpan BioSciences, Seattle, USA) and N-acetyl-β-D-glucosaminidase (NAG) (Jiancheng, Nanjing, China) levels in urine were both detected by enzyme-linked immunosorbent assay.

### Measurement of lipid accumulation in kidneys

Kidney tissues were fixed with 4% paraformaldehyde, dehydrated with 30% sucrose solution, and embedded into optimal cutting temperature compound. For oil red O staining, 0.2% oil red O stock solution was diluted with double distilled water at a ratio of 3:2. After filtration, the oil red O working solution was used to stain 6 μm frozen sections of kidney for 30 min at room temperature. For Filipin staining, 6 μm sections were stained with 50 ng/mL Filipin (Santa Cruz, Dallas, USA) for 30 min at room temperature. Cell samples were fixed with 4% paraformaldehyde and stained with oil red O and Filipin. The results of oil red O and Filipin staining were examined by light microscopy and laser microscopy.

Quantification of intracellular free cholesterol in kidney samples and cells was performed. Briefly, samples were exposed to a solution of chloroform/methanol (2:1) to isolate lipids. After they were homogenized with an ultrasound equipment, the lipid phase was collected, dried in a drying baker and redissolved with 2-propanol containing 10% Triton X-100. Quantitative measurement of free cholesterol in the abovementioned solution was determined using the method previously described 26. The solid phase was dissolved in 1 mol/L sodium hydroxide solution for protein quantification. The concentrations of free cholesterol in samples were calculated with a standard curve and normalized against the total cell protein.

### Measurement of acetic acid concentration

Acetic acid stock solution at 1000 μM was prepared by dissolving standard acetic acid in ethyl acetate and was further diluted with internal standard solution to obtain 200 μM, 100 μM, 50 μM, 20 μM, 10 μM, and 5 μM acetic acid solutions containing internal standard. Then, 50 µl serum was added to an equal volume of internal standard solution (4-methylpentanoic acid), acidified with 10 µl concentrated hydrochloric acid and vibrated for 30 s. Acetic acid in samples was then extracted by the addition of 1 ml ethyl acetate. After homogenization with ultrasound equipment for 1 min, samples were centrifuged at 12500 rpm/min for 5 min. The organic phase of the samples was obtained, filtered with a 0.22 µm strainer and stored in an autosampler vial at -80 °C for gas chromatographic analysis. Gas chromatograph (Thermo Fisher, Waltham, USA) separation was performed with a DB-23 capillary column (30 m*0.250 mm, Agilent, Palo Alto, USA). A 1 µl sample was injected in the split mode at a ratio of 7:1 with helium as the carrier gas at a constant flow rate of 1 ml/min. A standard curve was constructed to analyse the concentration of acetic acid in the samples. The peak area of acetic acid in each sample was normalized to the peak area of the internal standard.

### Western Blotting

Total protein extracted from the renal cortex or cells was denatured for Western Blotting analysis. First, protein samples were loaded and separated by sodium dodecyl sulphate-polyacrylamide gel and then transferred to a polyvinylidene fluoride membrane. After treatment with blocking solution, the membranes were incubated with primary antibodies against LDLr, HMGR, CD36, CXCL16, and GPR43 overnight at 4 °C, followed by secondary antibodies for 1 h. The results were detected by an ECL Advanced™ system (GE Healthcare Bio-Sciences, Uppsala, Sweden).

### Cell culture and small RNA interference

Human kidney 2 (HK-2) cell is a cell line obtained by infusing human papilloma virus (HPV16) E6/E7 gene into proximal tubular epithelial cell 27. The cells were cultured as described in our previous study 23. To knock down the expression of GPR43 in HK-2 cells, specific siRNA was transfected in cells with RNAiMax reagent (Invitrogen, Carlsbad, USA) according to the manufacturer's protocol. The nonspecific siRNA sequence was used as a negative control.

### Statistical analysis

The results are expressed as the mean ± SD. Statistical analysis was performed using SPSS v23.0 or Prism 7.0 GraphPad Software. Statistical comparisons among multiple groups were analysed for signifycance by one-way ANOVA followed by Newman-Keuls, least significant difference or Dunnett-t post-tests. Correlation between variables was analysed by Spearman's R coefficient. *P*<0.05 was considered a significant difference.

## Results

### Depletion of gut microbiota alleviated tubulointerstitial injury in diabetic rats

**Figure 1:**
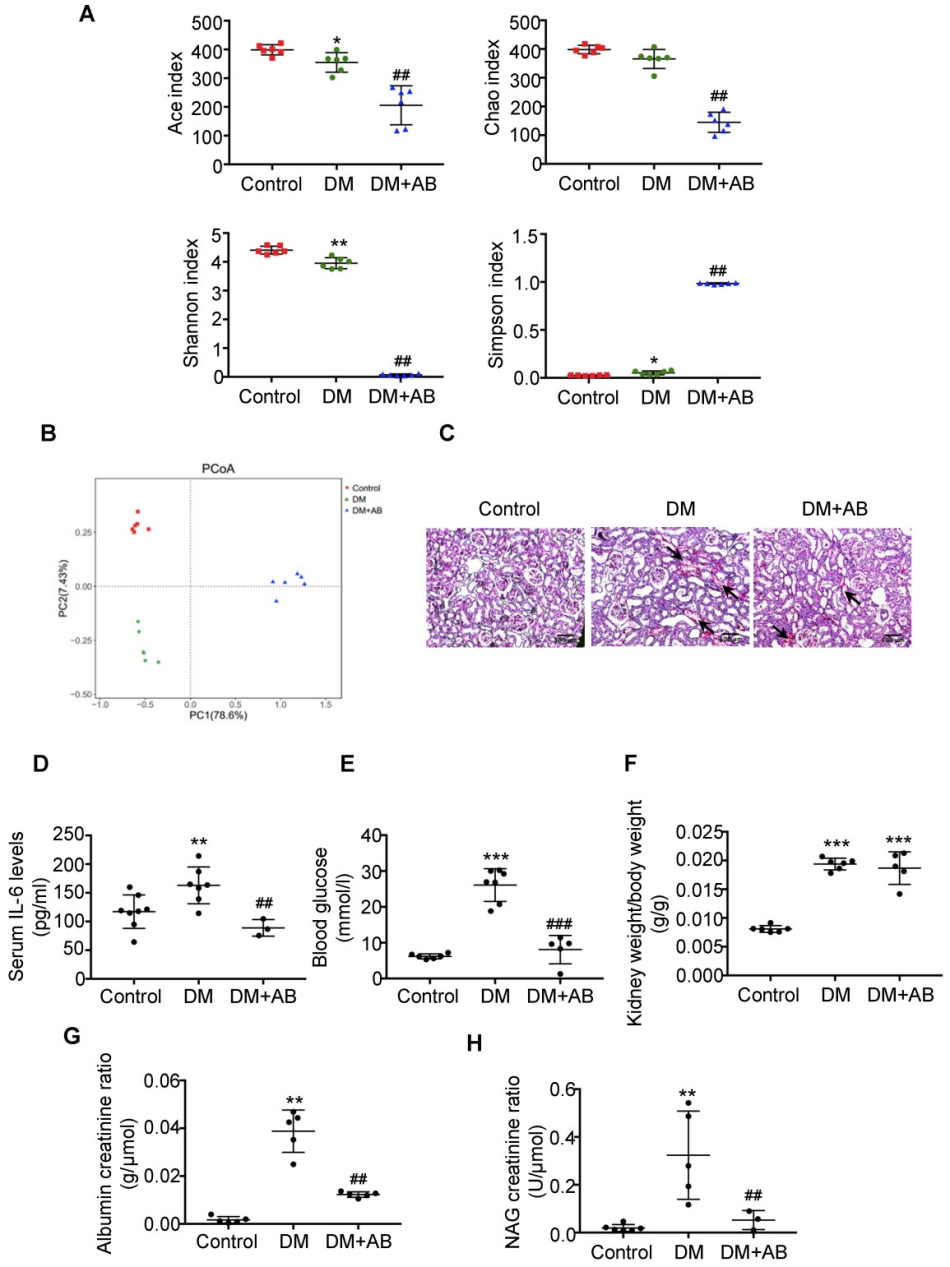
** Depletion of gut microbiota alleviated tubulointerstitial injury in diabetic rats.** Rats were divided into three groups: healthy controls (Control), diabetic rats (DM), and diabetic rats treated with a broad-spectrum antibiotics cocktail in drinking water (DM+AB). (A-B) Analysis of alpha diversity (Ace index, Chao index, Shannon index and Simpson index) and unweighted UniFrac-based principal coordinates analysis (PCoA) at the OUT level were performed to evaluate the abundances, diversity and composition of faecal microbiota in rats by 16S ribosomal DNA sequencing (n=6). (C) Histologic changes of the tubulointerstitium (shown by black arrows) were detected by PAS staining, original magnification ×100 (scale bar, 500 μm). (D) Serum levels of IL-6 (n=3-8). (E) Serum levels of blood glucose (n=5-7). (F) Ratio of kidney weight to body weight (n=5-6). (G) Urinary albumin creatinine ratio (n=5) and (H) urinary NAG creatinine ratio were measured (n=3-6). Data are expressed as the mean ± SD. ^*^P<0.05 versus the control, ^**^P<0.01 versus the control, ^***^P<0.001 versus the control, ^##^*P*<0.01 versus DM, ^###^*P*<0.001 versus DM. NAG, N-acetyl-β-D-glucosaminidase.

First, we analysed the abundances, diversity and structure of gut microbiota in stool samples by 16S ribosomal DNA sequencing. There was a significant decrease of bacterial abundance and diversity in diabetic rats compared with controls, and antibiotics treatment almost killed all the gut microbiota in the DM+AB group (Figure 1A). The composition and structure of gut microbiota in the three groups were different, as shown by unweighted UniFrac-based principal coordinate analysis (PCoA) at the operational taxonomic unit (OTU) level (Figure 1B). Histologically, PAS staining showed that there was extensive tubulointerstitial injury accompanied with tubular expansion and necrosis. Notably, depletion of gut microbiota treated by antibiotics significantly alleviated tubulointerstitial injury (Figure 1C). In addition, antibiotics administration decreased serum IL-6 level (Figure 1D) and effectively reversed elevated blood glucose levels in diabetic rats, although it had no effect on the ratio of kidney weight to body weight (Figure 1E-F). Meanwhile, increased urine albumin creatinine ratio and NAG creatinine ratio in diabetic rats were rescued by antibiotics treatment (Figure 1G-1H). These results indicated that depletion of gut microbiota by antibiotics administration protected against tubulointerstitial injury in diabetic nephropathy.

### Depletion of gut microbiota improved the dysregulation of cholesterol homeostasis in the tubulointerstitium

**Figure 2:**
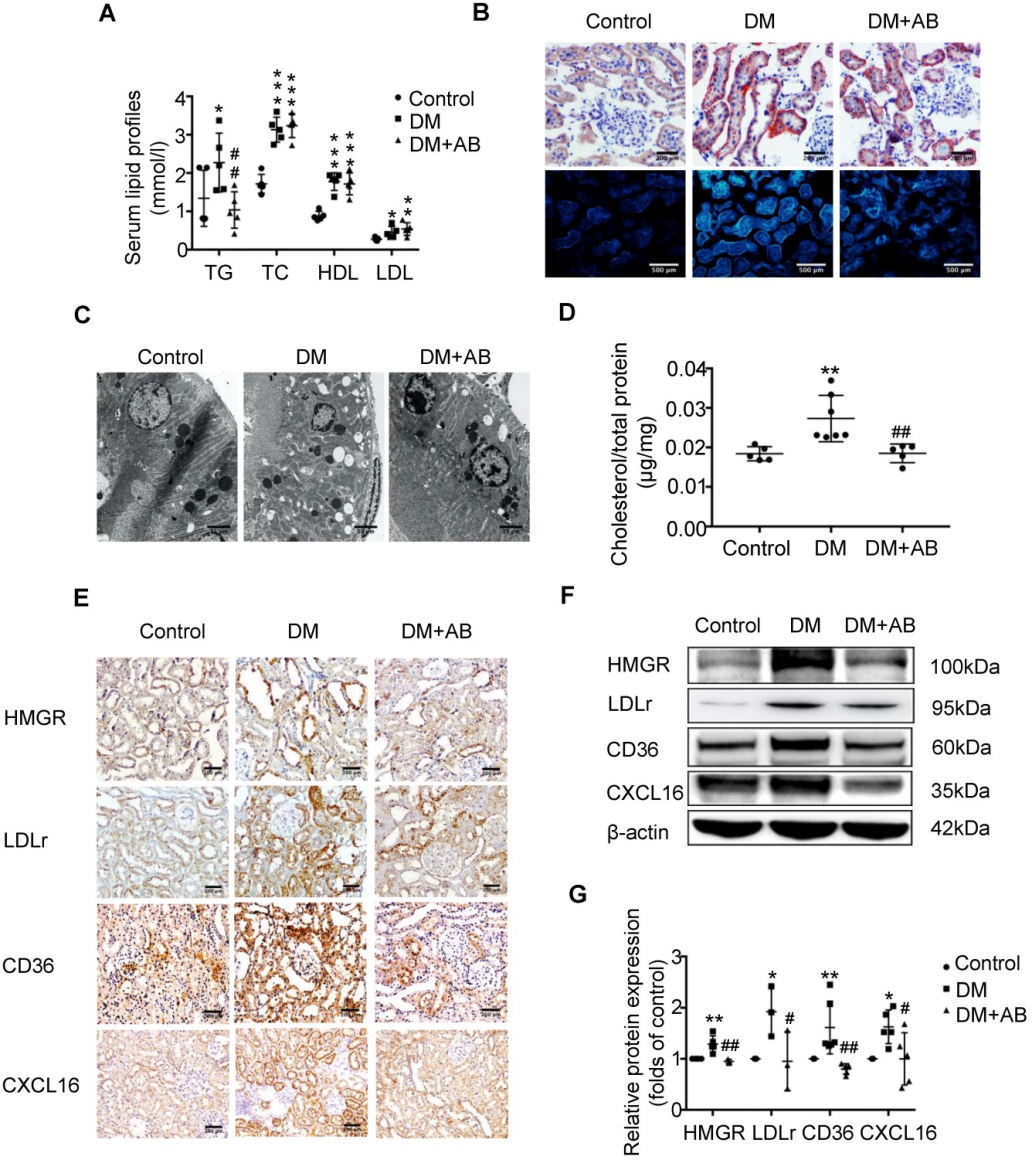
** Depletion of gut microbiota improved the dysregulation of cholesterol homeostasis in the tubulointerstitium.** (A) Serum lipid profile was analysed (n=5). (B) Oil red O staining (scale bar, 200 μm, original magnification ×200), Filipin staining (scale bar, 500 μm, original magnification ×200) and (C) transmission electron microscopy (scale bar, 25 μm, original magnification ×1500) were used to observe lipid accumulation in the tubulointerstitium. (D) Results of cholesterol content in the kidneys of rats was expressed as the ratio of cholesterol to total protein (n=5-7). (E) Protein expression levels of HMGR, LDLr, CD36, and CXCL16 in kidneys of rats were measured by immunohistochemical staining (brown colour, scale bar 200 μm, original magnification ×200) and (F and G) Western Blotting (n=3-6). The density of protein bands was quantified by software ImageJ, which was normalized by comparison with β-actin and expressed as a percentage of the control. Data are expressed as the mean ± SD. ^*^P<0.05 versus the control, ^**^P<0.01 versus the control, ^***^P<0.001 versus the control, ^#^*P*<0.05 versus DM, ^##^*P*<0.01 versus DM. TG, triglyceride. TC, total cholesterol. HDL, high density lipoprotein. LDL, low density lipoprotein. HMGR, 3-hydroxy-3-methylglutaryl coenzyme A reductase. LDLr, low-density lipoprotein, receptor. CXCL16, CXC chemokine ligand 16.

To investigate the potential mechanism of gut microbiota dysbiosis-induced tubulointerstitial injury, we measured serum lipid profiles and assessed lipid accumulation in kidneys. Serum levels of TG, TC, HDL and LDL were elevated in the DM group compared with the control; however, antibiotics treatment dramatically reduced TG levels in diabetic rats, although it had no effect on TC, HDL and LDL levels (Figure 2A). Oil red O staining and Filipin staining showed that there was significant lipid droplet accumulation in the tubulointerstitium of diabetic rats compared with controls, which was overridden by antibiotics treatment (Figure 2B). Ultra-microstructure analysis for lipid droplets assessed by transmission electron microscopy exhibited similar results (Figure 2C), which was further confirmed by cholesterol quantitative assays (Figure 2D). These results suggest that gut microbiota dysbiosis is involved in the dysregulation of cholesterol homeostasis in the kidneys of diabetic rats. Therefore, we further examined lipoprotein metabolism-related pathways. As demonstrated by immunohistochemical staining and Western Blotting, antibiotics treatment significantly reversed the elevated protein expression of HMGR, LDLr, CD36 and CXCL16 in the kidneys of diabetic rats (Figure 2E-G). This finding suggests that gut microbiota dysbiosis may mediate tubulointerstitial injury through the disruption of cholesterol homeostasis.

### Acetate produced from gut microbiota may induce the dysregulation of cholesterol homeostasis

**Figure 3:**
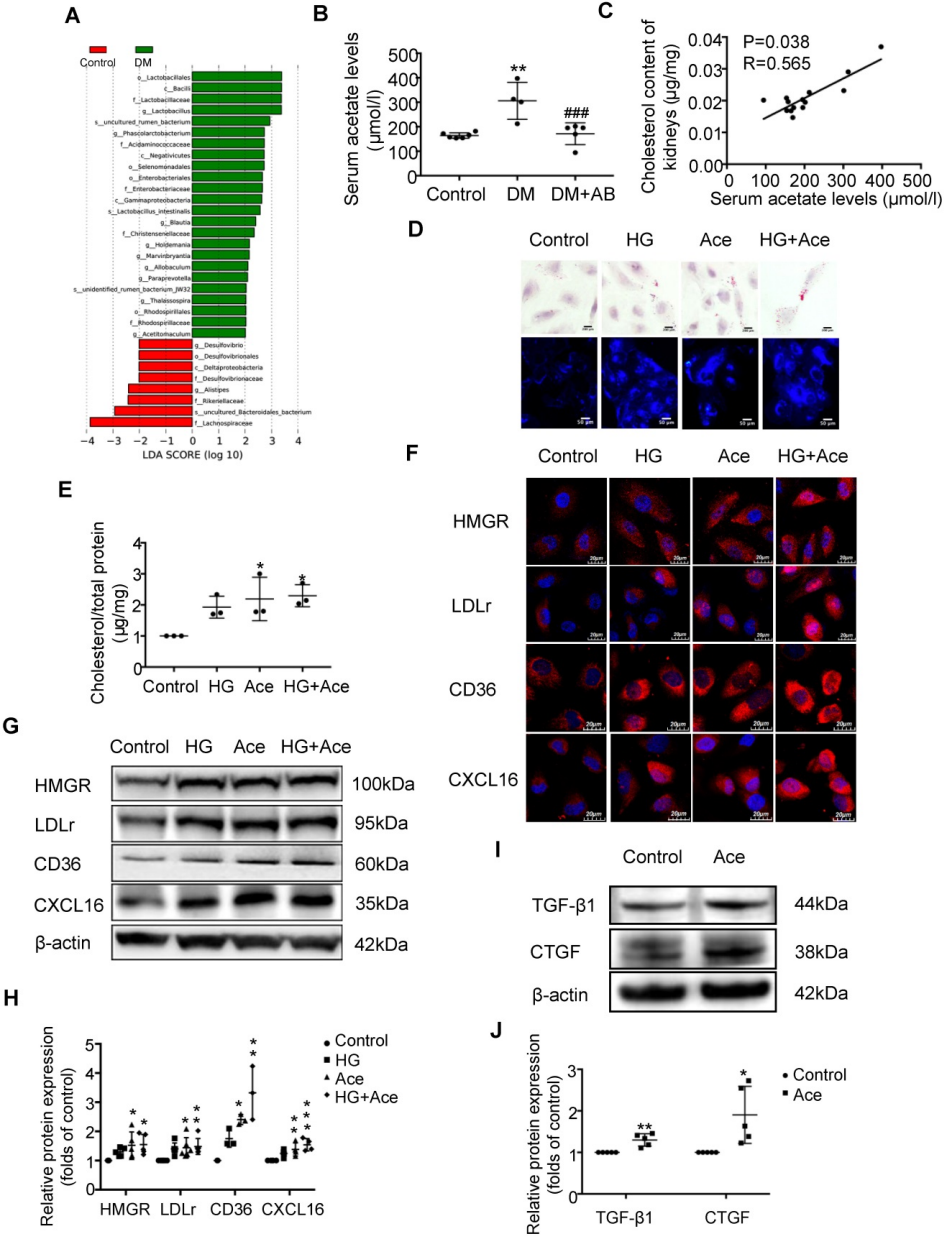
**Acetate produced from gut microbiota may induce the dysregulation of cholesterol homeostasis.** HK-2 cells were incubated with or without acetate (10 mM) in the presence or absence of glucose (30 mM) for 24 h. (A) Linear discriminant analysis (LDA) effect size (LEfSe) algorithm of OUT tables was used to determine taxa that best characterize each biological class in faeces of rats (n=6). (B) The serum acetate level of rats was measured by gas chromatographic analysis (n=4-6). ^**^P<0.01 versus the control, ^###^*P*<0.01 versus DM. (C) Correlation analysis between the serum acetate level and cholesterol content of kidneys was determined by Spearman's R coefficient (n=14). (D) Oil red O staining (scale bar, 200 μm, original magnification ×400) and Filipin staining (scale bar, 100 μm, original magnification ×200) were used to observe lipid accumulation in HK-2 cells. (E) Cholesterol content in HK-2 cells was quantified by free cholesterol quantification assays. (n=3 independent experiments). ^*^P<0.05 versus the control. (F) Protein expression of HMGR, LDLr, CD36, and CXCL16 in HK-2 cells were measured by immunofluorescence staining (scale bar, 20 μm, original magnification ×800) and (G and H) Western Blotting (n=3-6 independent experiments). ^*^P<0.05 versus the control, ^**^P<0.01 versus the control, ^***^P<0.001 versus the control. (I-J) Protein expression of TGF-β1 and CTGF was measured by Western Blotting (n=5 independent experiments). *P<0.05 versus the control, **P<0.01 versus the control. The density of protein bands was quantified by software ImageJ, which was normalized by comparison with β-actin and expressed as the percentage of control. Data are expressed as the mean ± SD. HMGR, 3-hydroxy-3-methylglutaryl coenzyme A reductase. LDLr, low-density lipoprotein, receptor. CXCL16, CXC chemokine ligand 16. TGF-β1, transforming growth factor-β1. CTGF, connective tissue growth factor.

We assumed that the metabolites produced from gut microbiota may circulate to the kidney and then induce the dysregulation of cholesterol homeostasis. Therefore, we first performed an analysis of the metabolite-producing gut microbiota. The results showed that there was a significant enrichment of acetate-producing *Lactobacillus* and* Phascolarctobacterium* in faeces samples of diabetic rats compared to the controls (Figure 3A). Accordingly, serum acetate levels in diabetic rats were markedly elevated (Figure 3B). Depletion of gut microbiota treated with antibiotics significantly decreased the serum acetate level of diabetic rats (Figure 3B). Interestingly, there was a weak but significant positive correlation between serum acetate level and cholesterol content of kidneys in rats (Figure 3C). Therefore, we further examined the effects of acetate on lipid deposition in HK-2 cells under high glucose conditions. Morphological observation and cholesterol quantitative assays both demonstrated that acetate significantly increased lipid accumulation in HK-2 cells (Figure 3D-E), which was correlated with the upregulated protein expression of HMGR, LDLr, CD36 and CXCL16 (Figure 3F-H). These results reveal that acetate induces lipid accumulation in HK-2 cells through the disruption of cholesterol homeostasis. Moreover, *in vitro* study showed that acetate treatment increased TGF-βand CTGF protein expression in HK-2 cells, suggesting that excessive acetate could be also involved in profibrotic effects on renal tubulointerstitium (Figure 3I-J).

### Faecal microbiota transplantation improved tubulointerstitial injury in diabetic rats

**Figure 4:**
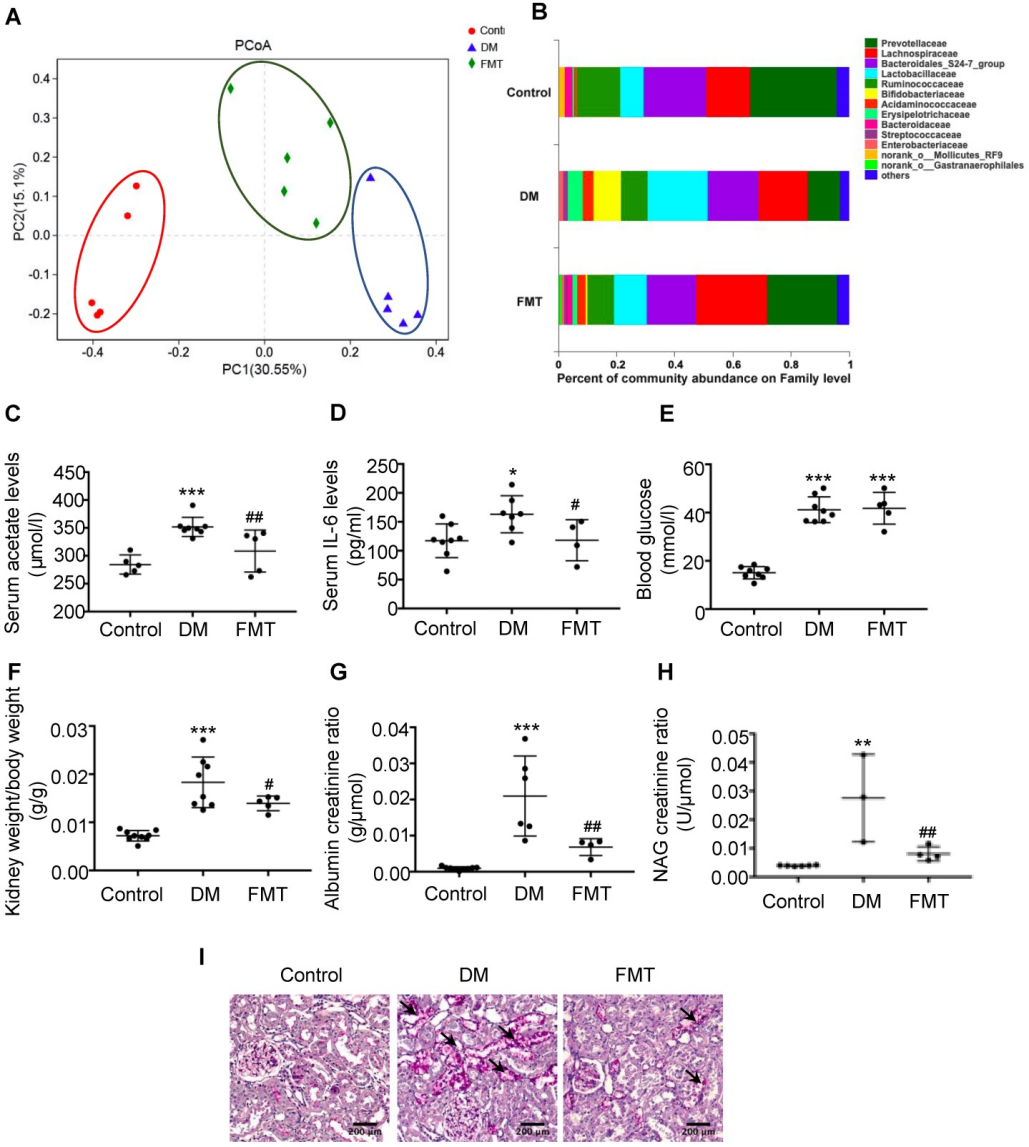
** Faecal microbiota transplantation improved tubulointerstitial injury in diabetic rats.** Rats were randomly divided into healthy controls (control), diabetic rats (DM), and diabetic rats transplanted with faecal microbiota extracted from control rats (FMT). (A) Analysis of unweighted UniFrac-based principal coordinates (PCoA) at the OUT level (n=5). (B) The percent of community abundance at the family level was determined to evaluate the composition of faecal microbiota in rats by 16S ribosomal DNA sequencing (n=5). (C) The serum acetate level of rats was measured by gas chromatographic analysis (n=5-8). (D) Serum levels of IL-6 (n=4-8). (E) Serum levels of blood glucose (n=5-8). (F) Ratio of kidney weight to body weight (n=5-9). (G) Urinary albumin creatinine ratio (n=4-9) and (H) urinary NAG creatinine ratio (n=3-6) were measured. (I) Histologic changes of the tubulointerstitium (shown by black arrows) were detected by PAS staining (scale bar, 200 μm, original magnification ×200). Data are expressed as the mean ± SD. ^*^P<0.05 versus the control, ^**^P<0.01 versus the control, ^***^P<0.001 versus the control, ^#^*P*<0.05 versus DM, ^##^*P*<0.01 versus DM. NAG, N-acetyl-β-D-glucosaminidase.

To further verify the effects of acetate on cholesterol homeostasis, we established a transplantation animal model of faecal microbiota from the control. Interestingly, the composition of gut microbiota in both the FMT group and the control was similar (Figure 4A). Furthermore, acetate-producing microbiota (*Lactobacillaceae*, etc.) was decreased in the diabetic group treated with FMT (Figure 4B), which was in accordance with the decreased serum acetate level (Figure 4C). FMT treatment did not affect the blood glucose level of diabetic rats (Figure 4E), whereas decreased serum IL-6 levels of diabetic rats (Figure 4D). The ratio of kidney weight to body weight, urine albumin creatinine ratio, and NAG creatinine ratio in the FMT group were decreased compared with the DM group (Figure 4F-H). PAS staining demonstrated that the desquamation and necrosis of tubular epithelial cells in the FMT group were alleviated compared with the DM group (Figure 4I). These results suggest that FMT effectively improves tubulointerstitial injury in diabetic rats.

### Faecal microbiota transplantation improved the dysregulation of cholesterol homeostasis in the tubulointerstitium of diabetic rats

**Figure 5:**
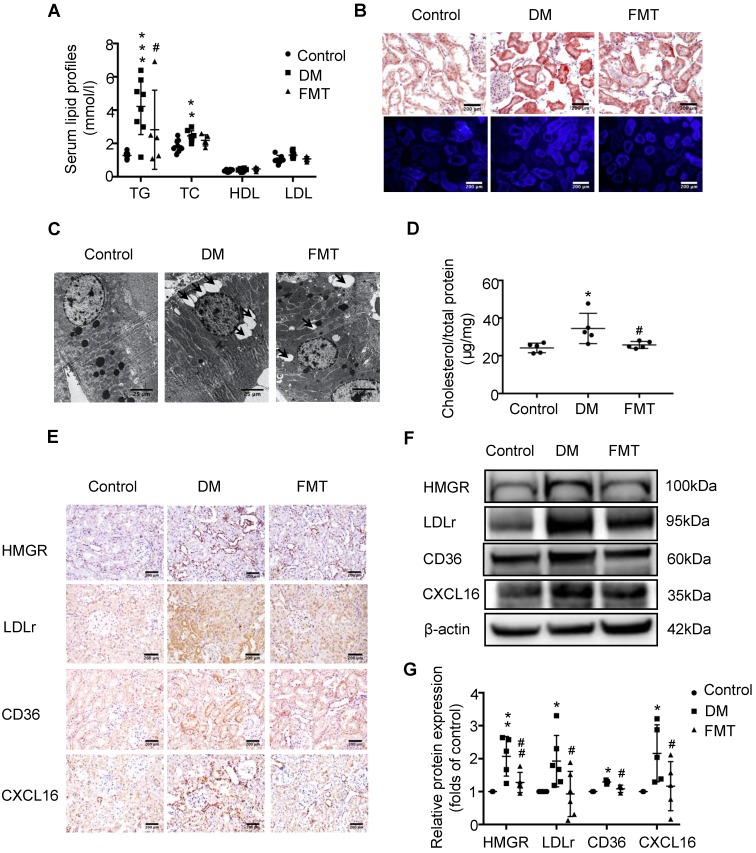
** Faecal microbiota transplantation improved the dysregulation of cholesterol homeostasis in the tubulointerstitium of diabetic rats.** (A) Serum lipid profiles was measured (n=5-8). (B) Oil red O staining (scale bar, 200 μm, original magnification ×200), Filipin staining (scale bar, 200 μm, original magnification ×200) and (C) transmission electron microscopy (scale bar, 25 μm, original magnification ×1500) were used to observe lipid accumulation in the tubulointerstitium. (D) Results of cholesterol content in the kidneys of rats was expressed as the ratio of cholesterol to total protein (n=5). (E) Protein expression of HMGR, LDLr, CD36, and CXCL16 in the kidneys of rats was measured by immunohistochemical staining (brown colour, scale bar, 200 μm, original magnification ×200,) and (F and G) Western Blotting (n=3-6). The density of protein bands was quantified by software ImageJ, which was normalized by comparison with β-actin and expressed as the percentage of control. Data are expressed as the mean ± SD. ^*^P<0.05 versus the control,^ **^P<0.01 versus the control,^ ***^P<0.001 versus the control, ^#^*P*<0.05 versus DM, ^##^*P*<0.01 versus DM. HMGR, 3-hydroxy-3-methylglutaryl coenzyme A reductase. LDLr, low-density lipoprotein, receptor. CXCL16, CXC chemokine ligand 16.

Next, we further examined the effects of acetate on cholesterol homeostasis. FMT markedly reduced the TG level in the serum of diabetic rats (Figure 5A). Moreover, FMT decreased lipid accumulation in the tubulointerstitium of diabetic rats (Figure 5B-D), which was mediated by the downregulation of the HMGR, LDLr, CD36 and CXCL16 pathways (Figure 5E-G). These results suggest that FMT improved tubulointerstitial injury in diabetic rats by overriding the disruption of cholesterol homeostasis.

### Acetate activated GPR43 to induce the dysregulation of cholesterol homeostasis

**Figure 6:**
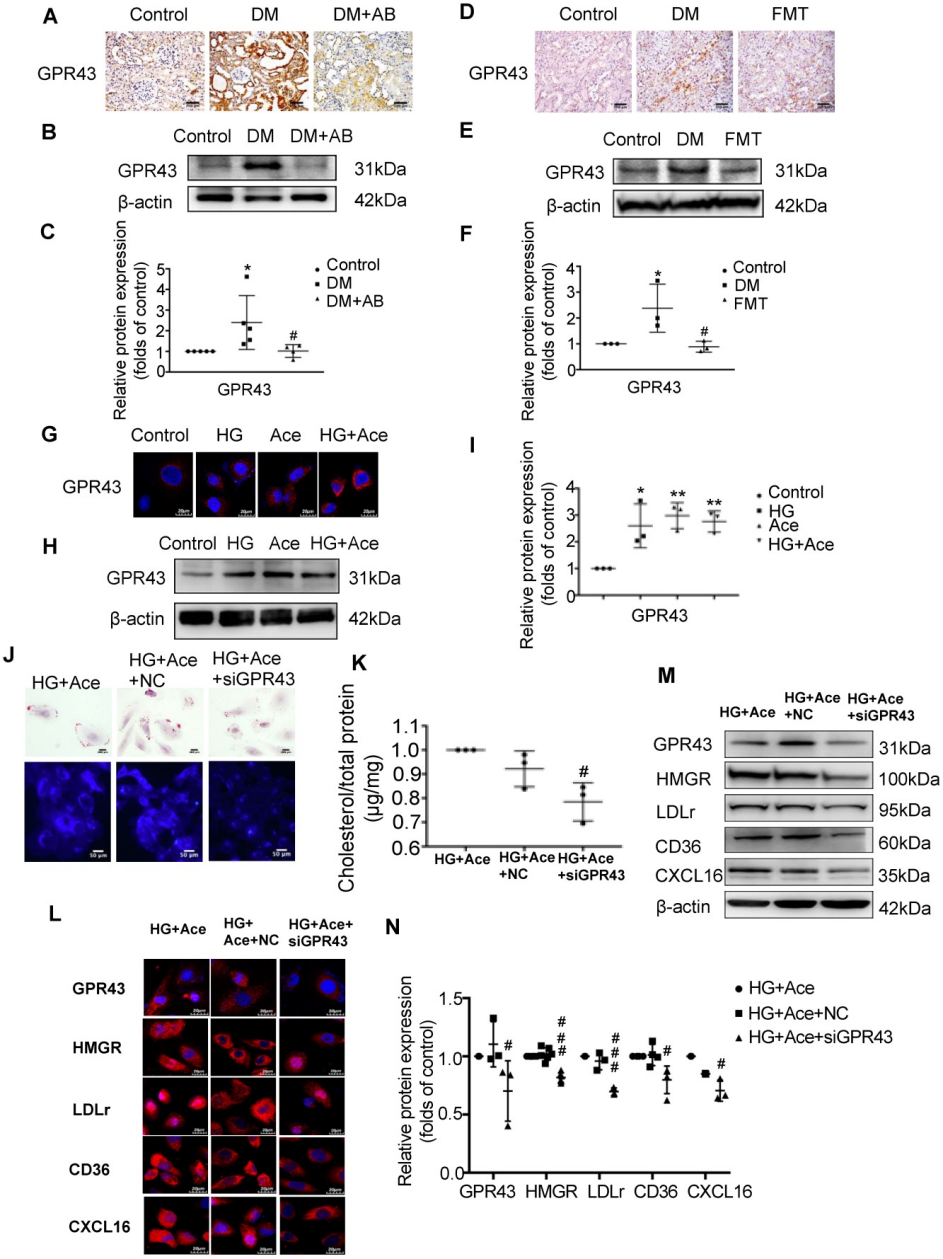
** Acetate activated GPR43 to induce the dysregulation of cholesterol homeostasis.** (A-F) Protein expression of GPR43 in kidneys of rats was measured by immunohistochemical staining (brown colour, scale bar, 200 μm, original magnification ×200) and Western Blotting (n=3-5). ^*^P<0.05 versus the control, ^#^*P*<0.05 versus DM. (G-I) Protein expression of GPR43 in HK-2 cells was measured by immunofluorescence staining (scale bar, 20 μm, original magnification ×800) and Western Blotting (n=3 independent experiments). ^*^P<0.05 versus the control,^ **^P<0.01 versus the control. (J) Oil red O staining (scale bar, 200 μm, original magnification ×400) and Filipin staining (scale bar, 100 μm, original magnification ×200) were used to observe lipid accumulation in HK-2 cells. (K) Cholesterol content in HK-2 cells was quantified by a free cholesterol quantification assay. (n=3 independent experiments). ^#^*P*<0.05 versus HG+Ace+NC. (L) Protein expression of GPR43, HMGR, LDLr, CD36, and CXCL16 in HK-2 cells was measured by immunofluorescence staining (scale bar, 20 μm, original magnification ×800) and (M and N) Western Blotting (n=3-6 independent experiments). ^#^*P*<0.05 versus HG+Ace+NC, ^###^*P*<0.001 versus HG+Ace+NC. Data are expressed as the mean ± SD. The density of protein bands was quantified by software Image J, which was normalized by comparison with β-actin and expressed as a percentage of the control. GPR43, G protein coupled receptor 43. HMGR, 3-hydroxy-3-methylglutaryl coenzyme A reductase. LDLr, low-density lipoprotein, receptor. CXCL16, CXC chemokine ligand 16.

We further examined the potential mechanism for the dysregulation of cholesterol homeostasis induced by acetate. We observed increased acetate levels in the serum and GPR43 expression in the kidneys of diabetic rats, whereas they were accordingly decreased by gut microbiota depletion and the FMT (Figure 6A-F). The results *in vitro* confirmed that acetate significantly increased GPR43 expression in HK-2 cells under high glucose conditions (Figure 6G-I). Further analysis showed that GPR43 siRNA treatment significantly reduced lipid accumulation in HK-2 cells even in the presence of acetate plus high glucose (Figure 6J-K), which was correlated with the decreased protein expression of HMGR, LDLr, CD36, and CXCL16 (Figure 6L-N). These findings suggest that activation of the acetate/GPR43 pathway may be involved in the dysregulation of cholesterol homeostasis.

## Discussion

Recent studies have reported that lipid metabolic disorders contribute to the progression of DN 22, 23, 28. However, the precise mechanism for lipid metabolism disorder-mediated kidney injuries in DN remains unknown. Some studies showed that gut microbiota dysbiosis is closely associated with the lipid metabolism disorder 29, 30. Therefore, this study aimed to investigate the effects of gut microbiota on the dysregulation of cholesterol homeostasis in the tubulointerstitial injury of DN. Our results preliminarily demonstrated that excessive acetate produced from gut microbiota disrupted cholesterol homeostasis to contribute to tubulointerstitial injury in DN.

Gut microbiota dysbiosis exists in diabetic patients. Qi *et al.* reported that the richness of faecal bacteria in child patients with type 1 DM was markedly lower than that in the healthy controls 31. Moreover, a study of 345 Chinese type 2 DM patients revealed that the abundance of gut butyrate-producing bacteria was decreased and accompanied by an increase in various opportunistic pathogens 32. Our study showed that there was a significantly decreased abundance of gut bacteria in diabetic rats compared with the controls. Moreover, the composition of gut microbiota was significantly different between these two groups, as indicated by PCoA. Interestingly, Fernandes *et al.* reported that gut microbiota dysbiosis contributes to the development of kidney diseases through the release of inflammatory cytokines and uremic toxins 33. Zhao *et al.* demonstrated that magnesium lithospermate B decreased the 24-hour urinary albumin level in diabetic mice by improving the dysbiosis of gut microbiota 34. In this study, we demonstrated for the first time that both gut microbiota depletion and FMT treatments alleviated tubulointerstitial injury in diabetic rats, characterized by improved desquamation and necrosis of tubular epithelial cells and tubular expansion. These findings suggest that gut microbiota dysbiosis may play important roles in contributing to tubulointerstitial injury of DN.

Recently, Zhou *et al.* found that the decreased serum levels of TG and TC in mice fed a high-fat diet and high-dose genistein were thought to be associated with the increased abundance of *Rikenella* and *Rikenellaceae_RC9* 35. Yin and colleagues demonstrated that melatonin alleviated high fat diet-induced adipose accumulation and improved gene expression of lipid metabolism in mice through reprogramming gut microbiota 36. Kasahara *et al.* demonstrated increased plasma cholesterol levels in germ-free mice, which was caused by reduced bile acid synthesis, suggesting that gut microbiota may be involved in cholesterol-derived bile acid synthesis 30. Our study showed that there was significant lipid accumulation in the tubuleinterstitium of diabetic rats compared to the controls. However, both the gut microbiota depletion and FMT treatments markedly decreased the TG level in the serum and lipid deposition in the tubulointerstitium of diabetic rats. Our data indicated that gut microbiota dysbiosis may disrupt cholesterol homeostasis to induce tubulointerstitial injury of DN.

Gut microbiota mainly participates in the progression of metabolic diseases through its metabolites. Canfora *et al.* 37 reported that products derived from microbial carbohydrate and protein fermentation were correlated with obesity and obesity-associated insulin resistance, type 2 DM and non-alcoholic fatty liver disease. Wollam *et al.* 38 showed that the microbiota-produced *N*-formyl peptide fMLF promoted obesity-induced glucose intolerance in high-fat diet-induced obese mice. Kikuchi *et al.* 39 demonstrated that gut microbiome-derived phenyl sulphate contributed to albuminuria in diabetic kidney disease. Our study showed that serum acetate levels in diabetic rats were markedly increased compared with the controls. However, both the gut microbiota depletion and FMT treatments significantly decreased serum acetate levels in diabetic rats, accompanied by decreased lipid accumulation in the tubulointerstitium mediated by accordingly decreased expression of LDLr, HMGR, CD36, CXCL16, which was further confirmed by acetate stimulation in HK-2 cells *in vitro*. These findings suggest that gut microbiota may overproduce acetate to disrupt cholesterol homeostasis. Moreover, our study demonstrated that GPR43 siRNA treatment significantly decreased acetate-induced lipid accumulation in HK-2 cells, accompanied by accordingly downregulated expression of LDLr, HMGR, CD36, and CXCL16. These results indicated that gut microbiota may disrupt cholesterol homeostasis through the activation of acetate/GPR43 signalling.

Acetate is one of the main components of short chain fatty acids (SCFAs). At present, there are some controversial opinions about the roles of SCFAs in health and diseases. Andrade-Oliveira *et al*. demonstrated that intraperitoneal injection with SCFAs, twice individually at 30 minutes before ischemia and the moment of reperfusion, improved acute kidney injury (AKI) by decreasing inflammatory cytokines and chemokines locally and systemically though inhibiting NF-kB signaling pathway 12. Yang *et al*. revealed that dietary fiber supplement significantly reversed kidney injuries in CKD mice which is associated with the increased SCFAs production from microbial fermentation 40. However, not all the treatment of SCFAs showed beneficial effects. Valproic acid administration does not reduce the accumulation of monocytes, macrophages and neutrophils in the angiotensin II-treated mice 41. SCFAs administration at higher than physiological levels for 6 weeks resulted in T cell-mediated ureteritis by inducing effector (Th1 and Th17) and regulatory T cells 42. Moreover, Zumbrun *et al*. reported that mice fed with a high-fiber diet got increased gut butyrate production and exhibited more susceptible to infection with *Escherichia coli* 43*.* Our study indicated that increased acetate production in diabetes contributed to the tubulointerstitial injury and albuminuria. We speculated that these discrepancies of SCFAs were possibly due to different animal models in different diseases and also correlated with the type, concentration, and application time of SCFAs.

Taken together, our study demonstrated that excessive acetate produced from gut microbiota disrupted cholesterol homeostasis to contribute to tubulointerstitial injury in DN through the activation of GPR43.

## Abbreviations

AB: antibiotics
AKI: acute kidney injury
CKD: chronic kidney disease
CTGF: connective tissue growth factor
CXCL16: CXC motif chemokine ligand 16
DM: diabetes mellitus
DN: diabetic nephropathy
FMT: faecal microbiota transplantation
GPR43: G protein coupled receptor 43
HDL: high density lipoprotein
HK-2 cells: human kidney 2 cells
HMGR: 3-hydroxy-3-methylglutaryl coenzyme A reductase
LDL: low density lipoprotein
LDLr: low density lipoprotein receptor
NAG: N-acetyl-β-D-glucosaminidase
OTU: operational taxonomic unit
PAS: periodic acid-schiff
PCoA: principal coordinate analysis
RAS: renin-angiotensin system
SCFAs: short chain fatty acids
STZ: streptozocin
TC: total cholesterol
TG: total triglyceride
TGF-β1: transforming growth factor-β1
